# The Role of Global Longitudinal Strain in Detecting Early Left Ventricular Dysfunction in Pediatric Bicuspid Aortic Valve Patients

**DOI:** 10.3390/children12060736

**Published:** 2025-06-06

**Authors:** Mohammed A. Mashali, Isabelle Deschênes, Carl V. Leier, Nancy S. Saad

**Affiliations:** 1 Department of Physiology and Cell Biology, Dorothy M. Davis Heart and Lung Research Institute, College of Medicine, The Ohio State University Wexner Medical Center, Columbus, OH 43210, USA; isabelle.deschenes@osumc.edu; 2 Department of Surgery, Faculty of Veterinary Medicine, Damanhour University, Damanhour 22514, Egypt; 3 Department of Internal Medicine, Division of Cardiovascular Medicine, College of Medicine, The Ohio State University Wexner Medical Center, Columbus, OH 43210, USA; carl.leier@osumc.edu; 4 Department of Pharmacology and Toxicology, Faculty of Pharmacy, Helwan University, Cairo 11795, Egypt

## 1. Introduction

Global longitudinal strain (GLS), assessed via speckle tracking echocardiography (STE), is increasingly recognized as a sensitive and early indicator of left ventricular (LV) dysfunction in pediatric patients with bicuspid aortic valve (BAV). In this editorial, we discuss the findings of the recently published pilot study by Făgărășan et al. [1], which appeared in *Children* as part of the Special Issue “*Heart Failure in Children and Adolescents*”, and explore the broader implications of segmental strain abnormalities and phenotype-specific evaluation for risk stratification and long-term monitoring. We also outline future directions in pediatric cardiac imaging and emphasize the need for multi-center studies to validate GLS as a prognostic tool in routine pediatric cardiology practice.

As one of the most common congenital heart defects, BAV often remains clinically silent in its early stages while predisposing patients to significant cardiovascular complications over time [2]. It is frequently associated with structural abnormalities, including aortic stenosis (AS), aortic regurgitation (AR), coarctation of the aorta (CoA), and progressive aortopathy [3,4]. Although many pediatric BAV patients remain asymptomatic, disease progression can result in substantial morbidity and eventually necessitate surgical intervention [5]. Traditionally, left ventricular ejection fraction (LVEF) has been the standard for evaluating LV function; however, it often appears normal even in the presence of early myocardial dysfunction [6]. Incorporating advanced imaging techniques alongside conventional echocardiography is crucial for detecting subtle myocardial impairments that may carry long-term consequences [7].

## 2. Global Longitudinal Strain as an Early Marker

The study by Făgărășan et al. [1] sheds light on an essential yet underexplored aspect of BAV in the pediatric population—the subtle LV dysfunction that may precede clinical symptoms and measurable declines in ejection fraction. In this prospective pilot study, the authors evaluated 73 pediatric patients with BAV using STE to assess GLS, comparing their findings to an age-matched control group of healthy children. They present compelling evidence that GLS is a more sensitive marker for subclinical myocardial dysfunction than the traditional LVEF. This finding enriches the existing literature, suggesting that conventional echocardiographic parameters may not adequately capture early myocardial changes [8,9,10,11], positioning GLS as a vital tool for risk stratification and early intervention in pediatric cardiology practice.

## 3. Segmental GLS Patterns and Personalized Risk Stratification

A critical finding of this study is the reduction in GLS within specific myocardial segments—particularly in the inferior and apical four-chamber views and the anterior segment. These subclinical myocardial alterations, despite normal global LV function, align with the existing research indicating that abnormal strain patterns can serve as early markers of myocardial fibrosis and contractile dysfunction, often preceding overt heart failure symptoms [10,12,13,14]. The early detection of these abnormalities allows clinicians to implement targeted monitoring and interventions, potentially slowing disease progression.

Furthermore, this study highlights the importance of phenotypic characterization in BAV patients. The IB phenotype was the most prevalent and frequently associated with mild AR. This supports previous research indicating that certain BAV phenotypes carry a higher risk of progressive valvular dysfunction and aortic dilatation [15,16]. Given these findings, personalized risk assessment based on BAV phenotypes can enhance clinical decision-making and patient management.

This raises important questions about clinical care strategies and long-term monitoring. Evaluating whether strain analysis should be incorporated into routine BAV assessments and whether early pharmacological or lifestyle interventions could provide cardiovascular benefits is essential. Additionally, longitudinal studies are needed to determine whether reduced GLS in pediatric BAV patients predicts increased morbidity in adulthood and whether early detection can improve long-term health outcomes.

## 4. Future Directions in Pediatric Cardiac Imaging

Future multi-center studies with larger cohorts and extended follow-up periods are essential to validate these findings and establish standardized clinical guidelines. Such studies would help confirm the broader applicability of GLS as a diagnostic tool. Additionally, addressing variability across imaging platforms will be crucial to ensure consistency and reliability in GLS measurements across diverse clinical settings.

While the findings of Făgărășan et al. are compelling, it is important to interpret them within the context of the study’s limitations. As a single-center pilot study with a modest sample size and an absence of longitudinal follow-up, its generalizability is limited. Moreover, the study does not fully account for potential confounding variables such as imaging variability, patient comorbidities, and clinical heterogeneity. Therefore, although GLS demonstrates promise as an early marker of myocardial dysfunction, its routine integration into pediatric BAV management should be approached with caution until it is validated by larger, prospective multi-center studies with long-term outcomes.

## 5. Conclusions

Făgărășan et al.’s study provides valuable insights into the early detection of myocardial dysfunction in pediatric BAV patients, reinforcing the role of GLS as a more sensitive marker than LVEF. These findings highlight the need for further research to validate GLS as a prognostic tool and integrate it into routine clinical practice, ultimately supporting early intervention strategies to mitigate disease progression and enhance long-term cardiovascular health in pediatric BAV patients.

## Abbreviations

The following abbreviations are used in this manuscript:

AR	Aortic regurgitation
AS	Aortic stenosis
BAV	Bicuspid aortic valve
CoA	Coarctation of the aorta
GLS	Global longitudinal strain
LVEF	Left ventricular ejection fraction
LV	Left ventricular
STE	Speckle tracking echocardiography
